# Restorative Resection of the Rectum—Its History and Present Status
*Part of a paper delivered to the Bristol Medico-Chirurgical Society on 13th January 1960.


**Published:** 1961-01

**Authors:** C. Patrick Sames

**Affiliations:** Consultant Surgeon, Bath Clinical Area


### RESTORATIVE RESECTION OF THE RECTUM—ITS HISTORY AND PRESENT STATUS*

BY

C. PATRICK SAMES, M.S., F.R.C.S.

*Consultant Surgeon, Bath Clinical Area*

The question of cancer and its cure is exercising many minds, both medical and lay. Speaking in most general terms, the rectum is one of the more favourable sites for carcinoma. This is largely owing to the inherent nature of the pathology of rectal cancer—the nature of its cellular division as compared for instance with that occurring in the stomach, and its accessibility to medical treatment. The rectum is a site where the principles of cancer surgery can be applied without difficulty; these are excision, *in one piece*, of (i) the growth, (ii) the organ in which it is arising, and (iii) the field of lymphatic drainage. But in this particular case the patient is left with a permanent colostomy.

Dr. Cuthbert Dukes (1958) whose work on the pathology of this condition is well-known, has pointed out that in early cases (with the disease confined to the bowel wall and with no spread beyond) the five-year expectation of life is 81·2 per cent. He points out that after making allowances for intercurrent deaths from other causes the corrected five-year survival rate rises to 97·7 per cent. Surely if we make any further advances it would almost seem as though the development of a carcinoma and its early and satisfactory treatment would be an insurance in favour of a few extra years, over and above those normally allotted to us!

To many, a permanent colostomy may seem a high price to pay for the hope of cure. A patient faced with this possibility, even though acquainted with all the true facts, cannot but be filled with understandable dismay. His natural abhorrence of an artificial opening in the bowel quite often results in the understandibly plaintive cry—"Is there *no* alternative"?

There are some surgeons who, in their earnest desire for the best radical treatment, tend to belittle the burden to be borne by the patient with a colostomy, and who consider that the sufferer, not having specialized knowledge, is in no position to judge the rights and wrongs of the case. In the final analysis this is of course true, and recognition of such a fact places a heavy responsibility on the surgeon. He must not be blind to the unpleasant disabilities attendant upon a colostomy, with all its inconveniences and potential accidents—accidents so shocking as, in the words of Kipling, "To milk the heart of a man, and shame him before his kind". The surgeon must weigh fully the maximum chances of eradicating the disease against the recognized disability of a colostomy. He must, in addition, make allowances for the average expectation of life of the patient. The surgeon may even have to consider the wisdom of a colostomy in the light of other disabilities; I once had to deal with a man who was not only blind, but had lost the use of his right arm. Therefore, although to strive to eradicate the disease and to leave a naturally continent patient is a worthy aim, it must be stated that many eminent authorities consider the hopes of cure and of sphincter preservation to be incompatible. Nevertheless, operations on the rectum with functional preservation of the sphincters might be said to have arrived—not

*Part of a paper delivered to the Bristol Medico-Chirurgical Society on 13th January 1960.

with a bang—but rather like a train groping its way through the fog to Temple Meads Station. It is interesting to speculate why such operations were so slow to be accepted.

HISTORY OF OPERATIONS FOR RECTAL CANCER

In the earlier attempts at operation for rectal cancer, the attack was made from the perineum with ablation of the lower rectum and anal canal, leaving the patient with a fistulous track to the outside through the granulation tissue. Later, attention was directed towards restoring continuity of the bowel by end-to-end suture after a cuff excision of the growth. The approach was a posterior one with excision of the coccyx and varying amounts of the lower sacrum. This became known as the classical Kraske operation, although there followed numerous modifications, mainly based upon the amount and shape of the excised sacral segment. These operations were disappointing, not only because of failure to cure, but because of the morbidity so often following a breakdown of the suture from tension of the upper segment and subsequent purulent fistula formation. Nevertheless some encouraging experience with this operation caused Grey Turner (1926) to be sympathetic towards sphincter-saving operations. During a discussion at the Royal Society of Medicine in 1948, he stated that seven out of fourteen patients had survived for over five years. The accounts of these seven patients make intriguing reading, for the majority seemed to be alive at that time, from eight to twenty-six years after the operation.

In the early days the failure of the Kraske operation led to attempts at establishing a terminal colostomy in the sacral region, and efforts were made to improve continence either by inserting a variety of bungs or stoppers, or by passing the colostomy through the fibres of the gluteus maximus or lumbar muscles. However it was soon found that these efforts failed, and it was better to have an incontinent abdominal colostomy than a doubtfully continent one in the sacral or lumbar regions. With the advancing safety of intraperitoneal surgery and an appreciation of the importance of the upward lymphatic spread, greater lengths of rectum and lower colon were excised by the perineal route after a preliminary left iliac loop colostomy had been established. This became the popular perineal excision, as advocated by Lockhart Mummery, an operation seldom done now, although in the elderly and infirm it might still be considered advisable. Its disadvantage was the restricted excision of the lymphatic field, but in addition convalescence was often prolonged because of the slow healing of the granulating perineal wound and the occasional persistent mucous fistula; and colicky pains occurred years later, owing to inspissation of cellular débris in the blind loop below the colostomy.

In 1908 Ernest Miles introduced his abdomino-perineal excision, leaving the patient with a permanent terminal iliac colostomy. The operation was based upon study of the lymphatic spread, and on experience of recurrences in the pelvis and perineum in patients treated by the earlier types of operation. Miles was a master craftsman, and a virile exponent of his views. He trained a long and loyal line of assistants who champion his operation to this day. With its 50 per cent over-all 5-year cure, this operation has become the yardstick by which all other forms of therapy are judged. The perineo-abdominal excision as advocated by Gabriel (1934) and practised also by Grey Turner, is in essence the same operation in reverse. But for the objection that it required multiple repositioning of the patient during the operation, it was based, to my mind, on sound arguments, and it has surprised me that it was never more popular. Perhaps it was the lack of euphony in its name that lost it some of its would-be adherents. However, with the exception of a few staunch adherents to each of these operations, the question was resolved, in about 1939, by the introduction by Lloyd-Davies and his colleagues of the "synchronous-combined operation", in which two surgeons worked simultaneously, one attacking the perineum and the other the abdomen. It is not the purpose of this paper to go into the merits and demerits of this operation, but there is little doubt that its major advantage over all previous procedures is the increased facility it offers when dealing with growths on the border line of operability. Its major disadvantage is the divided responsibility it entails. If the ureter is cut, which surgeon is to blame? It has, however, become the standard operation in most clinics. It leaves the patient with a terminal colostomy, and is based, as was the original Miles operation, upon the lymphatic drainage of the rectum as judged by the carcinomatous spread in advanced or recurrent cases. The diagram originally appearing in Miles's book has been printed in nearly all subsequent works on the subject. This shows the three major lines of lymphatic spread, namely upwards along the inferior mesenteric vessels, laterally over the levator ani muscles to the iliac vessels, and downwards to the perineum and eventually the superficial inguinal lymph nodes. It was this lateral and downward spread which led Miles to advocate, not only ablation of the anus and anal canal, but as wide as possible an excision of peri-anal skin, the levatores ani, and soft parts. Nevertheless, Westhues (1930) in Germany, after extensive studies, has shown that lateral and downward spread only occurs when the lymphatics running upwards to the pre-aortic nodes *are blocked by growth.* This work was published again in 1934, and credit must be given to Pannett (1935) for bringing it to the notice of British surgeons.

### RESTORATIVE RESECTION

About this time Pannett, Grey Turner, and a few others commenced to practise excision of rectal growths with reconstitution of the bowel by anastomosis of the colon to the lower rectal stump; first by abdominal and sacral approach, and later by the abdominal approach alone, now known as "anterior abdominal resection". It must be emphasised that this was not a resurrection of the old cuff resections of Kraske, but an operation directed towards a cure by resection of an adequate length of colon and the adjacent upward lymphatic field. Although at times this operation is done in advanced cases as a palliative measure (and should, in fact, be always considered) it was originally designed as a curative operation and did not deserve the name "conservative resection" by which it is often called. American surgeons refer to such operations as "sphincter-saving", or "resection with conservation of the sphincters", although such terms are not strictly accurate, for they would include the Hartmann's operation, which is not intended. "Restorative resection" is the most desirable term, and one which is now widely used in Britain.

Pannett based his operation on the pathological findings of Westhues, and argued that preservation of the levators and the anal canal was justified, because either the case was curable in that the lymphatic spread upwards had not yet become blocked, or the way was already blocked and the case already incurable—in which case there was no point in undertaking a mutilating operation by removal of the lower rectum, anal canal, and sphincters. However, this argument was not widely acceptable and the operation did not at first achieve general adoption. There are, I believe, numerous reasons for this. Firstly, the dominating personality of the brilliant surgeon Ernest Miles coloured most thought where rectal cancer was under consideration. Secondly, there was a wide misunderstanding of the arguments upon which the restoration was based, hence its inaccurate labelling as "conservative resection". Thirdly the technical difficulties of the operation were greater than those of the quick total ablation in the more standard operations. There were also the theoretical fears of insufficient viability of the rectal stump to permit of suturing; the fear of pelvic cellulitis; the possibilities of fistula formation, disruption of the anastomosis, peritonitis, and stricture formation. Although such hazards are mainly theoretical, they were, and still are, thought to be so great as to warrant a preliminary colostomy, or at least a simultaneous colostomy at the time of operation. This necessity for staging the operations is used by some as a strong argument against such procedures. However, in actual practice, such hazards have been found to be largely theoretical, and with the advent of the slowly absorbed sulpha drugs and the antibiotics the procedure of anterior abdominal resection has become relatively safe, even without preliminary or simultaneous colostomy. The fact the Pannett was undertaking such resections without colostomy before the days of sulphonamides or antibiotics is a high tribute to his surgical skill. Fistula formation does occur, but is seldom serious, and can be largely avoided by attention to details of technique. Disruption of the proximal colon due to tension is obviously the result of faulty surgery, but is likely to occur in the short, fat patient. Since it has been shown that paracolic lymph nodes only become involved proximally when the main lymph channels are blocked, it is advisable, when the colon appears to be short, to lengthen it by division of the first sigmoid or left colic vessels within the vascular arch and to leave the arcades intact to nourish the bowel. This marginal step, associated with mobilization of the splenic flexure, will nearly always result in a sufficient length of colon being available. In advanced cases where lymph spread upwards is extensive a "flush tie" of the inferior mesenteric artery on the aorta is advisable. Even then it may be found that the descending colon is viable in about 50 per cent of cases (Goligher 1949), and if it is not, it may be possible to bring down the left half of the transverse colon to complete the reconstruction.

The post-operative convalescence after abdominal restorative resection is surprisingly smooth as compared with the more standard operations, and the immediate shock is appreciably less. There would appear to be much less chance of post-operative bladder troubles, probably because the bladder is prevented from falling backwards by the new "rectum".

However, restorative resection was not to win a place of honour without some further struggles. Recurrences of growth were reported to occur on or near the suture line—mainly due, it is thought, to spill and implantation of cancer cells. This has been largely overcome by refinements in technique. Loss of rectal sensibility and associated incontinence were also reported if too little rectum had been left. At about the same time, doubt began to be cast upon the validity of Westhues's work when applied to growths situated in the lower rectum, it being felt that lateral spread over the levator ani muscles may occur earlier than was originally thought. These two factors have led to a more careful selection of cases. Curative anterior resection is now reserved for growths occurring not less than 10 cms. from the anal verge, above the peritoneal reflection, and showing no gross local spread. Preliminary biopsy should also have shown the growth to be of low or average malignancy. Anterior resection also has a great place in palliation when there are obvious secondaries in the liver or elsewhere.

### RESULTS

Although not universally accepted, restorative resection has been practised now for a sufficient number of years for large centres to be in a position to make a statistical assessment of it as compared with other forms of treatment. Recent survival statistics compiled by Naunton Morgan (1957) at St. Mark's Hospital shows a 54 per cent over-all five-year survival rate, which is slightly higher than for similar cases treated without restoration, for which cases the figure was 46 per cent. Workers from other centres have given rather similar results. It is, of course, tempting to attribute the slightly higher figure to more careful selection of cases, restorative resection being practised for earlier and more suitable cases. In comparing results it must not be overlooked that the formation of a colostomy itself has a certain mortality attached to it, which cannot be disregarded in long-term follow-up. Perforation and death have been known to occur from injudicious wash-out; and even suicide within the first few weeks is not unknown. Lateral space obstruction is a well recognized hazard, as is small bowel obstruction due to adhesions of loops at the sites where the colostomy is made.

Incidentally, wider familiarity with this operation has led to its extended use for diverticulitis coli and endometriosis, and other non-specific granulomata. Provided the operation of restorative resection is reserved for the right type of patient, it would seem that it offers a chance of survival at least equal to that of an operation involving a permanent colostomy.

### REFERENCES

Dukes, C. E. and Bussey, H. J. R. (1958). *British Journal of Cancer*, **12**, 309.

Gabriel, W. B. (1934). *Lancet*, *ii*, 69.

Goligher, J. C. (1949). *Brit. J. Surg.*, **36**, 175.

Morgan, C. N. (1957). *Proc. Roy. Soc. Med.*, **50**, 56.

Pannett, C. A. (1935). *Lancet*, *ii*, 423.

Turner, G. Grey (126). *Proc. Roy. Soc. Med.*, **19**, 27.

Westhues, H. (1930). *Arch. Klin. Chir.*, **161**, 582.

